# Linoleic acid: Is this the key that unlocks the quantum brain? Insights linking broken symmetries in molecular biology, mood disorders and personalistic emergentism

**DOI:** 10.1186/s12868-017-0356-1

**Published:** 2017-04-19

**Authors:** Massimo Cocchi, Chiara Minuto, Lucio Tonello, Fabio Gabrielli, Gustav Bernroider, Jack A. Tuszynski, Francesco Cappello, Mark Rasenick

**Affiliations:** 1 “Paolo Sotgiu” Institute for Research in Quantitative & Quantum Psychiatry & Cardiology, L.U.de.S. HEI, Malta, Switzerland; 2 Department of Veterinary Medical Sciences, University of Bologna, Bologna, Italy; 3 UCC, University of Cork, Cork, Ireland; 4 Neurosignaling Unit, Department of Organismic Biology, University of Salzburg, Salzburg, Austria; 5 Department of Oncology, University of Alberta, Edmonton, Canada; 6 Department of Physics, University of Alberta, Edmonton, Canada; 7 Department of Biomedicine and Neuroscience, University of Palermo, Palermo, Italy; 8 Euro-Mediterranean Institute of Science and Technology, Palermo, Italy; 9 Department of Physiology & Biophysics and Psychiatry, University of Illinois College of Medicine, Chicago, IL USA; 10 Jesse Brown VAMC, Chicago, IL USA

**Keywords:** Mood disorders, Linoleic acid, Ion channels, Cytoskeleton, Microtubule, Lipid raft, Depression, Antidepressants, Ising model, Quantum states

## Abstract

In this paper we present a mechanistic model that integrates subneuronal structures, namely ion channels, membrane fatty acids, lipid rafts, G proteins and the cytoskeleton in a dynamic system that is finely tuned in a healthy brain. We also argue that subtle changes in the composition of the membrane’s fatty acids may lead to down-stream effects causing dysregulation of the membrane, cytoskeleton and their interface. Such exquisite sensitivity to minor changes is known to occur in physical systems undergoing phase transitions, the simplest and most studied of them is the so-called Ising model, which exhibits a phase transition at a finite temperature between an ordered and disordered state in 2- or 3-dimensional space. We propose this model in the context of neuronal dynamics and further hypothesize that it may involve quantum degrees of freedom dependent upon variation in membrane domains associated with ion channels or microtubules. Finally, we provide a link between these physical characteristics of the dynamical mechanism to psychiatric disorders such as major depression and antidepressant action.

## Background

The knowledge of the neuraxis (and, in particular, brain) anatomy and physiology is complicated by the fact that, still today, the heterogeneous population of neurons and glial cells is far from being properly characterized at a molecular level. Much attention, in the last decades, has been paid to genes and proteins, in terms of expression, subcellular localization and molecular pathways and trafficking, but many studies neglected to focus on another, ancestrally basic, component of the nervous cells, the lipids that not only form the barrier between the internal and external environment but also have a dramatic role in the homeostasis of the entire nervous environment.

Since 1929 when Burr and Burr demonstrated the characteristics of essentiality of linoleic acid for animal organisms, some studies have tried to correlate the intake of this fatty acid with health status and, in particular, have investigated the functioning of the brain. In reality the presence of a minimum concentration of linoleic acid in the brain, together with the demonstration of its negligible transformation into the longer-chain derivatives and the low significance of linoleic acid diet on fatty acid composition of the brain, have not allowed, in the past, relevant interpretive access to its possible functional complexity in the brain.

Several studies on the fatty acid composition of the brain of many animals have consistently recorded concentrations around 1% [1–4]. Cocchi et al. [5] have tried to explain such a reduced concentration of linoleic acid as a stabilization element of the membranes, recognizing that this property is at least partially due to its melting point (−5 °C) and its spatial dimension. Its melting point places it as an intermediate between palmitic acid (+63 °C) and arachidonic acid (−50 °C), that is, the extremes of the most significant fatty acid profile of the brain, conferring and maintaining an appropriate level of mobility of the membrane, for example, as occurs in extreme environmental conditions with regard to temperature [6, 7].

We offer as the most likely explanation for the extremely low concentrations of linoleic acid, in the normal terrestrial habitat, a fortiori, the essential role such a fatty acid could play as an element that controls a symmetry breaking effect (representing a bifurcation point for the system at which two qualitatively different global behaviors can ensue) for cell membranes in the neurons and glia. This situation, involving extreme sensitivity to small, even infinitesimal changes in the control parameter values, determining the system’s response in terms of its dynamical behavior, is well known in the physics of phase transitions. Near a critical state (also called a transition point) in systems undergoing phase transitions [8] a single physical control parameter (e.g. pressure, temperature, or relative concentrations of chemical species in a mixture), determines the nature of the equilibrium state of the physical system under consideration. A classic example involves a magnetic system composed of tiny magnets represented by spins arranged geometrically in a regular array. In principle they can be oriented in arbitrary directions but they also interact with each other via nearest-neighbor coupling which is called a spin–spin interaction. For this system, there is a competition between entropy that favors disorder in the spin orientations and spin alignment along one direction due to their spin–spin coupling energy, which is minimized for parallel alignment. At high temperatures, entropy “wins” and the system is disordered. At low temperatures, the opposite happens and the spins orient themselves along the same axis. The boundary between two ranges of temperature is called the critical point (or critical temperature) and close to a critical point very exotic behavior can be found where the system is infinitely sensitive to external stimuli such as magnetic fields and the dynamic behavior of the system’s constituent spins is correlated over long distances such as small perturbations propagate from one end to the other. The two stable phases that arise are called a paramagnetic (disordered, also called symmetric) phase and a ferromagnetic (ordered, also called broken symmetry) state.

By analogy with the above example, we propose that linoleic acid’s concentration in the membrane of neurons or glia acts as a control parameter (corresponding to temperature for magnetic systems), effectively behaving like a switch in a system close to criticality, i.e. resulting in a phase transition between a normal brain and a pathological brain when the linoleic acid concentration falls below its set value. It is important to emphasize that systems close to criticality exhibit long-range order which is important in this case so that an effect at a cell level can propagate macroscopically to the organ level, i.e. this is not just a local effect but a global effect that involves the entire brain. The question emerges, what would this change in concentration entail regarding the dynamics of the brain, in particular the functioning of a single neuron or astrocyte? Furthermore, it is important to understand the mechanics of such an effect exerting influence on the sub-neuronal constituents such as the cytoskeleton.

Recent observations, conducted on the characterization of the platelet fatty acids in mood disorders and ischemic heart disease have consistently shown that a very low concentration of linoleic acid is correlated with these diseases. This observation was found to be particularly interesting in brains of depressed subjects who had completed suicide. Moreover, it seem to be supported by the work of Lalovic et al. [9] for which, even if not significant, perhaps for a limited number of cases, it is noted that in the ventral prefrontal cortex (a decision making area in humans) of depressed or bipolar suicidal subjects there is a slight decrease of linoleic acid, as supported by a standard deviation of the data, compared to the control groups. Together with this observation, also the results achieved of a very low concentration of linoleic acid in platelets of subjects with suicide attempts, have encouraged researchers to consider whether, in fact, it was possible to record small and significant variations of linoleic acid in other circumstances. For this purpose, a research group from the School of Agriculture and Veterinary Medicine (University of Bologna) [data not published] has recently evaluated the fatty acid compositions of the brain of obese rats. In fact, in the brains of the obese rats, there was a statistically significant reduction of linoleic acid [10].

The set of variations in the concentration of linoleic acid in the brain, undoubtedly, becomes interesting from the perspective of interpretation regarding its position in the membrane and the likelihood that these minor changes can result in the connections that the membrane makes to the cytoskeleton through the interactions with the G protein, Gsα and ion channels. A mechanistic model of this interaction could be built starting with the fatty acids in the membrane forming an ordered network whose structure depends on the specific composition of the fatty acids, in particular the linoleic acid. To embark on developing such a model we first discuss how lessons from the physics of phase transitions can help us understand the possible consequences for neuroscience.

## Predictions from the Ising model

In fact, our proposal is an extension of an already existing extensive body of literature in membrane biophysics where the lipid composition of the membrane has been modeled in terms of the two-dimensional Ising model [11]. The Ising model is a standard approach to the modeling of critical phenomena in physics. This model predicts a transition between a disordered and an ordered phase of a material as a function of temperature or another control parameter which is coupled to the order parameter describing the gross features of the system. For magnetic systems, an order parameter is total magnetization and a control parameter is temperature. In the case of membrane implementations of the Ising model, the ordering structure involves the tails of the phospholipids composing the membrane so the order parameter could be the average membrane thickness for example while the control parameter could be the fatty acid composition of the membrane, for example the percentage amount of linoleic acid.

As mentioned above, a 2D Ising model is a classical example of a system exhibiting a phase transition between ordered and disordered state when the temperature is lowered below a critical point [12]. For the benefit of the reader, we explain the key features of the model in order to better understand how it can be made useful in applications to brain dynamics. The total energy of this type of magnetic system is calculated using the so-called Hamiltonian function, namely:

1 $$H = - J\varSigma s_{i} s_{j} - B\varSigma s_{i}$$

where *J* is the interaction constant, *s*_*i*_ is the spin, whose value can be either −1/2 or 1/2, for the *i*th magnetic particle in the spin lattice, *i* and *j* are indices labeling the adjacent particles and B is the magnitude of the externally applied magnetic field that can be used to align spins along its direction irrespective of the temperature. In the absence of the external field B, the transition to an ordered state occurs at a well-defined temperature given by the interaction strength constant *J*:

2 $$T_{c} = 2.269J/k_{B}$$

**Fig. 1:**
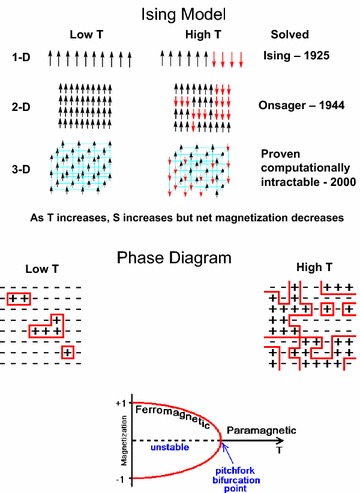
A schematic illustration of the Ising model and the symmetry-breaking spin ordering phenomenon below a characteristic temperature T_c_. The *left panel* shows the ordered arrangement of spins at low temperatures while the *right panel* shows the disordered spins at high temperatures. Higher entropy, S, and lower net magnetization are associated with spin disorder and vice versa

This temperature, called the critical (or Curie) temperature has major significance because of the nonlinear behavior of the system at and near this value. The system exhibits extreme sensitivity to parameter changes when it approached the critical temperature from either above or below. Importantly, the nature of the spin ordering in the Ising model depends on spatial dimensionality (no transition above the absolute zero temperature occurs in the 1D case, for example), which is shown in Fig. 1. This figure demonstrates graphically the nature of ordering taking place in the system near criticality and some associated properties. This phenomenon exists in all dimensions except for linear systems (1D) but the values of the transition temperature change whether it is a 2D or a 3D system.

It is important to note that the Ising model is simply a blueprint for a physical effect in a system that can spontaneously reorganize itself when a specific parameter is varied. At criticality, the system is shown to be bistable, i.e. it can choose one of the two possible orientations for the ordering of the spin variables (all spins pointing up or all spins pointing down; with equal probability).

How can this be translated into the dynamics of membrane’s phospholipids? Earlier work [11, 13] represented the orientation of the double bonds in fatty acid tails either as trans or cis, which can be mapped on the spin variable in the Ising model. In other words, we could consider a spin-up state as trans and a spin-down state as cis. Furthermore, instead of the temperature change causing an order–disorder transition in magnetic systems, one can introduce a parameter that defines a relative concentration of one type of fatty acid over another. In our case, the control parameter that drives the transition would be the linoleic acid concentration, whose critical value drives the system towards a phase transition causing a re-ordering of the double bonds in the membrane’s fatty acids in the same manner as temperature approaching the critical value causes the spins in a magnet to correlate their orientations over large distances. In the case of a fatty acid organization of the membrane, this ordering of fatty acid tail states can additionally cause a mechanical rigidity change of the membrane as well as a major effect on the functioning of the ion channels due to the electro-mechanical membrane-protein coupling [14]. Below we discuss how this interaction can provide a link to quantum biology aspects of the functioning of neurons.

## The quantum Ising model and the interactions between membrane fatty acids and ion channels

Interestingly, 1D and 2D Ising models have been also employed to describe the cooperative effects between ion channels in the membrane [15]. This means that ion channels operate synchronously and their activity can be controlled by the state of the membrane, in particular its rigidity and electrostatic trans-membrane potential that directly couples to the electric charge of the ions. This mechanism could bring a completely new perspective on the interpretation of quantum effects in the brain, which have been debated ever since the work of Penrose was published [16, 17]. At the most fundamental level the argument for the need of quantum physical nature of the functioning of the brain has to do with non-algorithmic cognitive processes, which cannot be performed by classical computation. This led Penrose, Hameroff and others to speculate that the brain operates in the quantum regime [18, 19]. How quantization of mental processes happens nobody really knows but it has been hypothesized that at least some brain dynamics occurs via delocalized quantum wave function operation [20]. This mechanism would confer clear advantages in the operation of cognitive functions in the brain and could explain some amazing feats of the human brain such as incredibly short reaction times of some athletes to situational changes or the ability of some geniuses to solve problems by intuitive insights without breaking these problems down into algorithmic subroutines. Instead, quantum search algorithms could be used that almost instantaneously explore the entire space of available states.

**Fig. 2:**
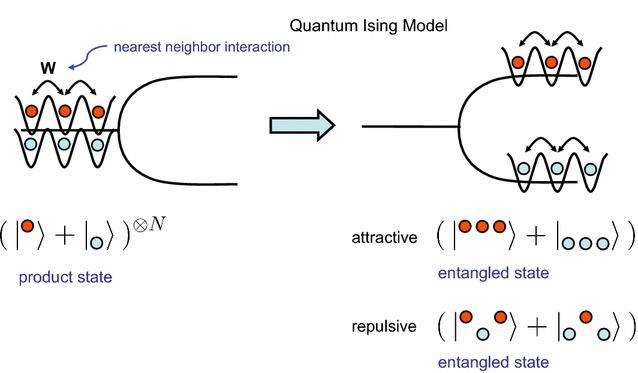
Ion tunneling in ion channels can be viewed as a basis for a quantum Ising model. A symmetric state on the *left* is a product state of ions occupying the two available quantum states marked *red* and *blue*. A broken symmetry state is shown on the *right* where the attractive and repulsive ionic arrangements are split into two distinct possibilities

Interestingly, a quantum Ising model has been exactly solved mathematically and its solution leads to the existence of long-range correlations and wave function entanglement in a process that Penrose and his colleagues speculated about in connection with brain dynamics [21]. However, in the present proposal, we postulate that the quantum coherence built by these interactions involves ions collectively passing through ion channels in the neuronal membrane, as a specific implementation of the quantum Ising model. As discussed above, the membrane’s composition in terms of fatty acids can be viewed as acting as a control parameter, which could induce a classically ordered state (or not, depending on the linoleic acid’s concentration). Hence the linoleic acid composition is as important as temperature in magnetic systems (obviously in human physiology temperature is kept fairly constant so it cannot play a critical role in the brain). This fatty acid organization, subsequently couples to the ion channels that can operate at a quantum level as we briefly discuss below. Note that in view of the above discussion, we predict that the fatty acid ordering phenomenon is described by a classical Ising model (where the variables have two real values: trans or cis, just like spins +1/2 or −1/2), while the ion channel dynamics is described using the quantum Ising model where the variables are quantum wavefunctions because ions can have a probabilistic distribution functions describing their location. The latter aspect has been recently elaborated on in mathematical detail by other authors [23, 24]. Figure 2 schematically illustrates how the tunneling of ions can form a basis for a quantum Ising model.

Furthermore, there may be a difference in hydrophobicity among different fatty acids of the brain and this may produce significant variations of the fatty acids dynamic of the phospholipid bilayer of neuronal membrane [22]. This could affect the decoherence times of quantum excitations in the collective states of ions traversing the ion channels and in turn affect mental processes. Decoherence times define the duration of a quantum state before it manifests itself by a so-called wave function collapse meaning its actual value can be measured or observed. A quantum state can be a superposition state so that two or more specific states coexist. This can be metaphorically illustrated by having simultaneous thoughts of ordering sushi and pasta before making a decision. Once we order sushi, this is akin to the wave function collapse and making an actual choice. Decoherence time is the time it takes to collapse a quantum state. If it’s too short, the system operates almost always classically. On the other hand, if the decoherence time is too long, quantum states could become too long-lived or trapped, and in the case of brain dynamics they may lead to obsessive thoughts and behaviors.

In a nutshell, what we suggest regarding the role of linoleic acid in this context is its effect on the ordered state of the cell membrane, which in turn affects the (possibly quantum) dynamics of the ion channels in neurons. Ion channels exhibit tunneling phenomena that have been described by quantum mechanical Hamiltonians and hence offer a strong link between possible quantum effects in the membrane and in the cytoskeleton [23, 24]. There may be an additional connection to the functioning of the cytoskeleton as we describe below. This is very important in view of the involvement of the cytoskeleton in neuronal information processing as well as a host of neurodegenerative diseases [25].

## Zones of signal convergence: the interaction between ion channels and the cytoskeleton

Electrical signaling in the brain builds on the concerted translocation of charges through integrated membrane channel proteins. Ever since the determination of the atomic resolution structure of channel proteins by MacKinnon et al. [26, 27], a highly detailed biophysical picture of the dynamics underlying the function of these proteins has been established. In particular, it was demonstrated that ion channels support quantum coherence resulting in characteristic resonances for the opening and closing of ion channel gates [23, 24].

Having outlined a possible connection between a detailed composition of the neuronal membrane and the functioning of ion channels, the next question to address is how this interaction can be further transmitted to the cytoplasm and the cytoskeleton. A connection to the cytoplasm can be readily provided by actin filaments that interact with the cell membrane [28]. There may also be a feedback mechanism operating in the opposite direction where the cytoskeleton affects the dynamics of the plasma membrane [29, 30]. This latter effect is closely associated with G proteins that act as shuttles between the membrane and both actin and tubulin filament networks [30].

**Fig. 3:**
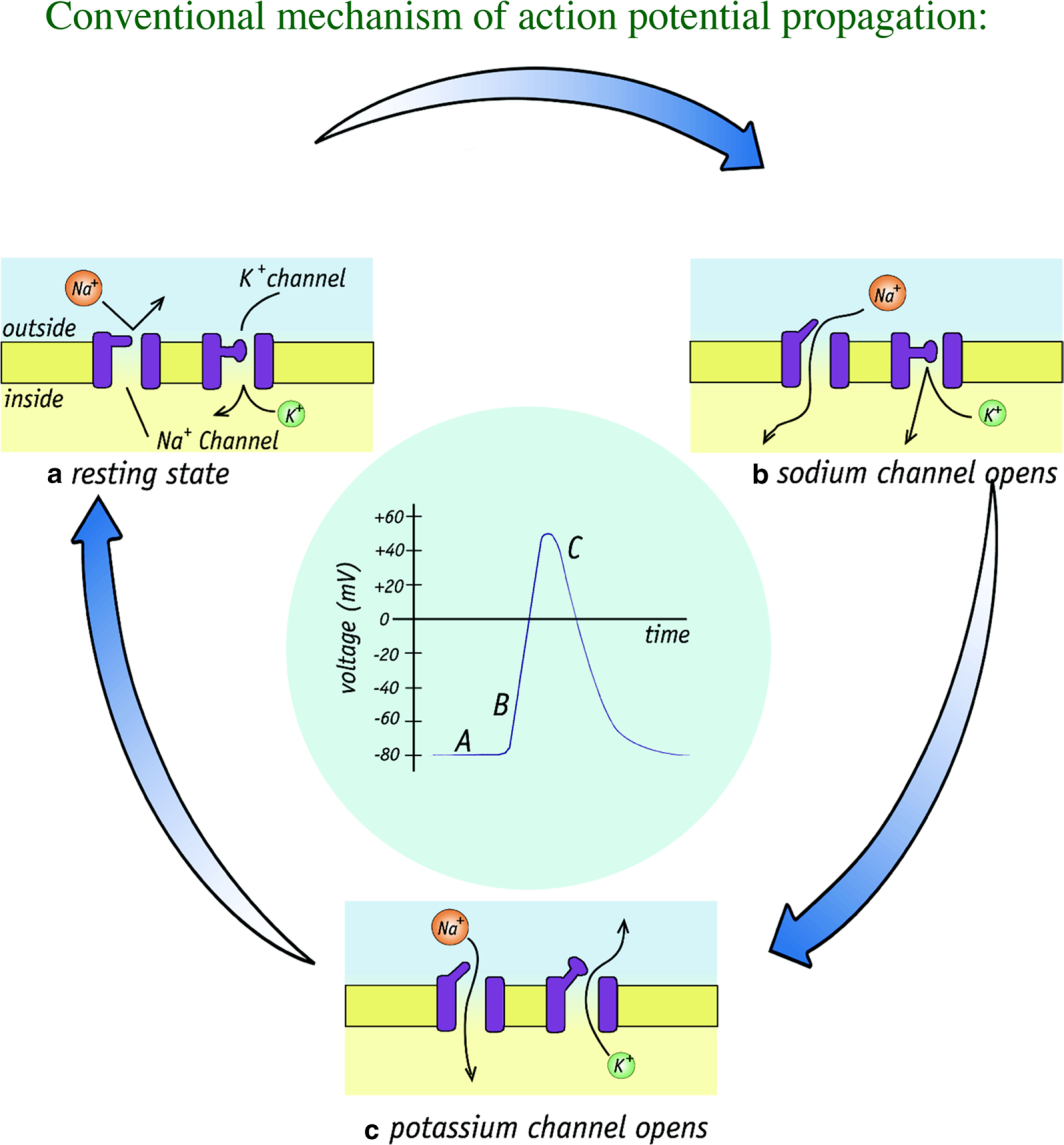
Schematic illustration of the standard representation of action potential propagation in neurons

The conventional mechanism of action potential involves the Hodgkin–Huxley model shown schematically in Fig. 3 and it just involves the membrane crossing ion fluxes propagating in and out of the cell via ion channels.

**Fig. 4:**
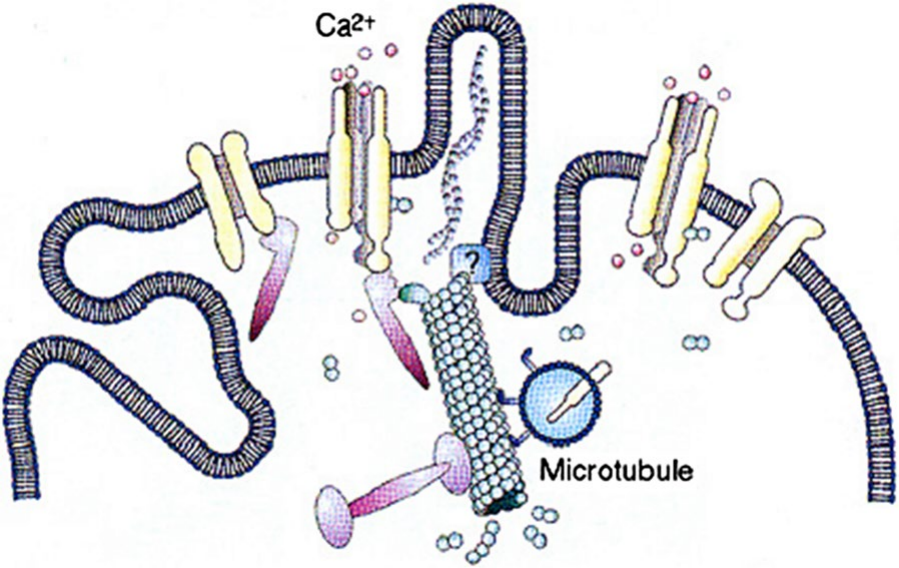
A depiction of the microtubule interaction with calcium ion channels in the membrane and potentially directly with the membrane. A microtubule is shown depolymerizing at the minus end and providing a track for a kinesin molecule movement toward the plus end. The symmetric purple structure attached to the microtubule is a microtubule associate protein (MAP) while the asymmetric purple structures interacting with the ion channels and indirectly with the microtubule are KIF3B molecules

However, there is ample evidence that the cytoskeleton is involved in the action potential regulation, which would indicate a bidirectional communication between ion channels and the cytoskeleton as suggested above. Microtubule depolymerizing agents (e.g., colchicine) eliminate peripheral cytoskeleton and action potential together [31, 32] and modify G protein signaling in mammalian cerebral cortex [33]. Microtubule stabilizing agents restore cytoskeleton and action potential together [31, 32]. Divalent ions such as magnesium and zinc restore cytoskeleton and action potential, according to Tasaki et al. [34]. On the other hand, calcium ions destabilize microtubules and actin filaments directly or indirectly by interactions with the various associated proteins. Consequently, the filaments of the cytoskeleton are likely to undergo highly localized reorganization in response to ion fluxes through the ion channels. Neurotransmitters, working through GPCRs can also alter microtubule dynamics, leading to changes in morphology of dendritic spines and the synapses located thereon [35, 36]. Conversely, microtubules and actin filaments may affect the opening and closing rates of ion channels. This has been extensively described in the work of G. Pollack [37] and is schematically illustrated in Fig. 4.

**Fig. 5:**
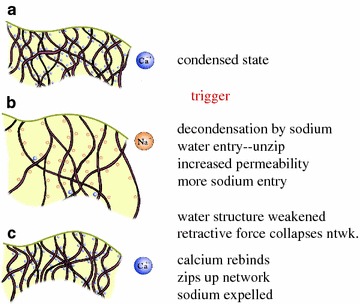
Effects of calcium and sodium ion concentration changes affecting condensation and decondensation of cytoskeletal protein networks

Following on Pollack’s hypotheses, we believe that the cell’s major communication systems (secretion, action potential generation) are governed by phase-transitions (symmetry breaking phenomena) taking place first in the structure of the membrane fatty acids, which are communicated to the negatively charged polymers of the cytoskeleton (actin filaments and microtubules) that are initially condensed by divalent cations. Then, monovalent cations (Na, K) trigger filament a decondensation transition due to ionic flows (see Fig. 5). This cytoskeletal transition is reversible as it depends on the ionic concentration. Note that the work of Rasenick et al. [35] suggests activated Gsα is internalized through lipid rafts where it activates tubulin GTPase on the plus end of the microtubule, increasing microtubule dynamics and plasticity of synaptic structures such as spines.

It is interesting to consider the action of anesthetics in this connection. It is well known that they eliminate the propagation of action potentials. General anesthetics span a broad range of molecular shapes and sizes hence fail to fit into the “lock and key” paradigm. They exhibit no covalent chemistry but bind to hydrophobic regions of proteins and membranes. Anesthetics are also known to form water clathrates [38]. In a recent paper [39] anesthetics were shown to interact with numerous binding sites on tubulin in addition to an earlier demonstration of binding to actin filaments. Consequently, anesthetics may exhibit multiple modes of action: direct binding to membranes that inhibits action potential propagation as well as binding to the cytoskeleton that may indirectly affect the cell’s membrane. Moreover, according to Pollack, they *could also* inhibit action potential by preventing required water uptake into the cell [37].

One might also consider the relationship of antidepressants to the lipid microenvironment. Antidepressant compounds (save ketamine) require an extended period of treatment (up to 2 months) before they achieve clinical efficacy. Presumed targets of these drugs (monoamine transporters or catabolizing enzymes) are inhibited after a short treatment, so some other factor, perhaps an intrinsic property of the membrane, must also be implicated. Most known antidepressants have been shown to target lipid rafts [40] and this is a gradual process [41].

## The role of the cell membranes in psychopathology (neurons, glia and platelet)

Plasma membrane of neurons and glia offer a promising way towards a better understanding of psychopathology in its different aspects: Major Depression, Bipolar Disorder and their effect on cognitive impairment. Several approaches have provided evidence about this subject. Rasenick et al. [42] have demonstrated that, in brain membranes from depressed suicides, that Gsa is enriched in lipid raft fractions where it is less complexed with adenylyl cyclase. The observation of diminished Gs adenylyl cyclase coupling was also been demonstrated in platelets [43, 44] and leukocytes. Thus, is suggested that the degree of lipid raft association of platelet Gsα might serve as a biological marker for depression.

Cocchi and Tonello [45–47] have studied the membranes of platelets of depressed subjects, highlighting the fatty acid profiles as a possible measure of the state of membrane.

The results obtained with the use of an artificial neural network (Self Organizing Map), have highlighted the possibility of classifying Mood Disorders by the so-called normality and, inside of Mood Disorders, to distinguish, with extreme precision, the subjects with Major Depression (MD) from those with Bipolar Disorder (BD). The results of the authors’ experiments seem very consistent with those obtained on G proteins associated with –plasma membrane lipid rafts delineated by the group of Rasenick et al. [42] as well as with the observations that the antibipolar drugs lithium and valproate, do not behave in a manner similar to antidepressants with regard to mobility of Gsα from lipid rafts [48]. The cell membrane lives, therefore, a constant condition of mobility, and, when the oscillations of fatty acids cross the tolerance ranges of the same, can be expressed pathological phenomena.

The mechanical factors of membrane lipids constitute lipid dependent factors that can dramatically affect protein functions or protein–protein interactions [49] and result in pathological consequences [22]. The condition of constant mobility of the membrane, whether for physiological levels or trespassing in pathological consequences, results in a deformation of the same at different levels of intensity with consequences that involve the raft lipid membrane, the protein Gsα, the cytoskeleton and the ion channels [50].

A number of factors influence the relationship between membrane and cytoskeleton, including the lipid composition, the density and the distribution of the cytoskeleton, the ratio between the surface area and volume, the internal cell pressure [51]. In the literature, the responses to the changes induced by forces of the mechanical type on the cytoskeleton have been extensively described [52–60].

All acquired knowledge, the study of quantum hypothesis of consciousness, the opportunity for dialogue that is open within the Quantum Paradigm Psychopathology Group (QPP), has certainly contributed to the construction of a new attempt hypothesis that seems to reconcile the previous ones, which, in reality, are only the result of different ways of looking at the same phenomenon.

The state of continual renewal and exchange of fatty acids by the membrane with consequent deformation of the lipid bilayer, can be thought of in terms of a mechanical force, in both physiological and pathological conditions, which is exercised on the cytoskeleton with continuity allowing, when there are physiological conditions for the membrane, periods of decoherence of microtubules (500 ms), which give rise to the conscious manifestation which will result in classic neurocorrelate of consciousness. The problem that arises is to understand how, under different conditions of mood disorders, the decoherence can be modified by lengthening or shortening the period (< or >500 ms). Having had the opportunity to understand that a higher mobility of membrane characterizes MDD and lower, BD, it is perceived that there are pathological conditions of deformation of the membrane that may exert different mechanical forces on the cytoskeleton from the inside of the same membrane and could potentially affect the physiological phenomenon of superposition of microtubules and consequently the period of oscillation.

In the case of BD there would be a shortening of the period of decoherence while in the case of MDD, an elongation. This eventuality could correspond, in cases of extreme criticality of decoherence period, to deep and decisive changes in the conscious state [22].

Having shown that between MDD and BD there is a symmetry break [61], keeping in mind the characteristics of mobility of the membrane, that there is a “domain wall” governed by electromagnetic forces that are likely to be the only form of communication between the two phenomena (MDD and BD) and that the only commonality of the two conditions is suicide, it seems consistent to think that such an event can take place for critical lengthening or shortening of the periods of decoherence, and, therefore, of the capacity of consciousness. These neuron forces, or “neuromechanics”, could represent, the element conditioning the control domain of the microtubules and conditioning their relationship with the criticality of synapses, cortex and serotonin [62].

## Conclusions

Much experimental evidence and consistent assumptions argue for a critical role of linoleic acid in the cell membranes. We know that is possible to measure very small changes of the brain Linoleic Acid’s concentration in pathological conditions. What we cannot explain is what escapes to the phenomenology of events such as memory, consciousness, and even suicide. We are not able to understand the “sub microscopic level”. We can measure the concentration of linoleic acid but we are not able to understand the complexity of the phenomena that it sets up as an end controller of the molecular fine-tuning, when, in the dynamics of the elements that relate to the cell between inside and outside, it affects the consciousness of individuals and affects their pathological responses.

The interactome, that is defined “as the whole [array of] molecular interactions that take place in an organism and allow the cascade of regulatory molecules including the mechanism of action of enzymes and metabolic reactions”, it might respond to small, critical change in concentration of linoleic acid that are lost in the complexity of the quantum entanglement.

**Fig. 6:**
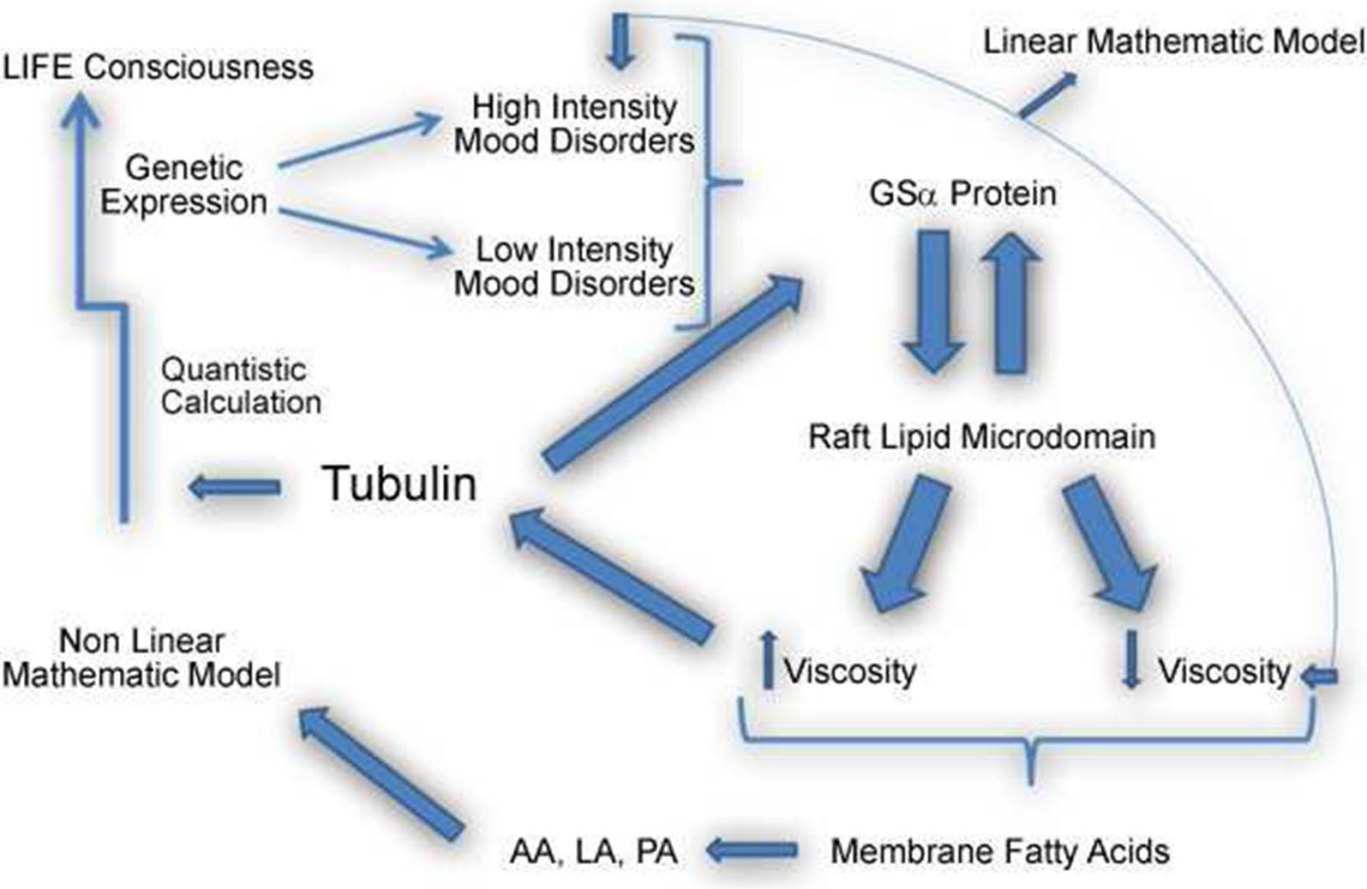
A schematic integrating the central role of tubulin interacting with Gsα proteins, lipid rafts and membrane fatty acids

In this case, beyond the possible connections among serotonin, platelets, cytoskeletal proteins and other molecular interactions with implications regarding the genesis of the brain behavior. Cell membrane and fatty acids, in particular linoleic acid is the “key that opens to the quantum brain” through the dynamics of the cytoskeleton. Our proposed integrated picture of the classical/quantum dynamics at the level of a neuron but including the cytoskeleton is shown in Fig. 6.

The approach to the processes of mind-consciousness, in their normal and pathological dynamics, cannot, however, settle on isolated elements and plans, observable by a privileged and aloof observer, but thinking and acting operationally, by connections, relationships, networks. All this, always in the belief that science is narration of a world of expressive, emerging conditions—for this never reducible to the sum of its elementary ingredients—and in which the symmetry breaking provide variety, creativity, vitality, as a sign of self-natural organization, of evolving systems, thanks to spontaneous breakage of symmetry, towards conditions increasingly complex and unpredictable, including the person as an *emerging relationship*.
